# Teaching in the Light of Activity Theory Applied to Preschool: Reflections on Brazilian Practice

**DOI:** 10.11621/pir.2023.0306

**Published:** 2023-09-30

**Authors:** Marcela Cristina de Moraes

**Affiliations:** a Federal University of Jataí, Brazil

**Keywords:** cultural-historical, activity theory, teaching practice, early childhood education, role-playing

## Abstract

**Background:**

Activity Theory applied by the teacher to preschool education favors the development of new psychological formations, such as perception, attention, memory, thought, language, and voluntary self-regulation, which prepare the child for school.

**Objective:**

To highlight the contributions of N.F. Talyzina based on Activity Theory applied to preschool education and to reflect on the theory’s use in the Brazilian education system.

**Design:**

This article is theoretically built from research in a sandwich doctorate program in Puebla, Mexico and internship supervision practices for psychologist training at a public university in Brazil’s central-west region.

**Results:**

Activity Theory is seldom applied to teaching, including in Brazil, and there is little knowledge about the scientific contributions of one of its practitioners, the late N.F. Talyzina. We chose the preschool stage as the focus of our reflections, and we maintain that the introduction of role-playing as the main activity in early childhood education promotes the development of psychological neoformations and prepares the child for the next stage of school. Finally, we present the internship practices in applied psychology in a Brazilian children’s group, with evidence of advances.

**Conclusion:**

There is a need for expansion of the formative experiments reported here to the Brazilian population, for scientific dissemination of the results, and promotion of teacher training and qualification in Activity Theory.

## Introduction

Activity Theory applied to teaching is rarely used in educational systems, being restricted to some centers of followers and researchers, including Kepler College (Colegio Kepler), in the city of Puebla, Mexico (Solovieva & Quintanar, 2015), where I had the pleasure of collecting data for my doctorate (Moraes, 2018) and evaluating the positive effects of using Activity Theory in preschool.

Nina F. Talyzina is little known in Brazil, and her theoretical contributions are not applied in the education system. The Studies and Research Group on Developmental Didactics and Teacher Professionalization (Grupo de Estudos e Pesquisas em Didática Desenvolvimental e Profissionalização Docente, GEPEDI) organized a series of publications to present to Brazilian readers the biographical profiles and contributions of Russian theorists linked to Marxist historical-cultural psychology. Among these are two chapters dedicated to N.F. Talyzina: “Aportaciones de N.F. Talizina para la psicologia y el desarrollo de la educación en el mundo y America Latina (“Contributions of N.F. Talyzina to psychology and the development of education in the world and Latin America) (Solovieva & Quintanar, 2015) and “Vias para la formación de la motivación escolar (Ways to form school motivation)” (Talyzina, 1923, trad. Pedrini & Malusá, 2017).

A survey on the CAPES portal, an important national database, pointed to increased publications in the last eight years about Activity Theory Applied to Mathematics Teaching, referencing Talyzina’s productions. Few works, however, pertain to the preschool stage.

Studies and experiments of Brazilian teaching practice are justified in order to expand the dissemination of the Activity Theory’s contributions to the preschool stage and its use in the educational system.

According to V. Davydov (1988), the concept of activity was initially introduced to psychological theory by L.S. Vygotsky (1896–1934), followed by great contributions by S.L. Rubinstein and A.N. Leontiev. At the end of the 1930s, Leontiev and Rubinstein began investigations of the formation and development of the psyche and consciousness and further developed the concept of activity. However, they differed in their understanding of activity and its relationship to the psyche, which led to different didactic systems.

One principle that explains the relationship between psychology and activity is that the human psyche is presented and constituted in activity (Rubinstein, 1986; Talyzina, 2009). People use their psyche to guide themselves and solve their problems. Interaction with the social environment, where problems are solved, is called an activity. With that in mind, the person

…participates as the active initiator, not a psyche recipient. The person performs not only external practical actions, but also psychic actions. The psyche is not only a picture of the world and a system of images, but also a system of actions (Talyzina, 2009, p. 14).

The individual subject reproduces historical-cultural forms of activity through internalization, by participating in the collective and socially organized realization of the activity; this activity thus becomes individual and internal (Davydov, 1988; Vygotsky, 2006).

One of Leontiev’s premises, supported by the *German Ideology* of Marx and Engels, is that people modify their thoughts when acting on the outside world. In this sense, “what people are is determined by their activity” (Leontiev, 1978, p. 21).

A person’s life is a set of successive activities, activity being the intermediate point between the material and the ideal, between the object and the mind. The activity’s role is to guide the person through the world of objects. However, it is important to highlight that this activity only has recognition and importance if it becomes part of collective life, subordinated to a system of social relations. Thus, individuals’ place in society and living conditions influence their activity (Leontiev, 2009).

Following Vygotsky’s guidelines, the investigator in a scientific study must work with the most elementary unit that carries all the characteristics and qualities of the analyzed phenomenon. In the case of activity, it is action that carries the whole (Talyzina, 2009).

Rubinstein (1989), cited by Talyzina (2009), states that action is the unit that carries the specificity of the activity, because activity and action have the same structure,

... goal, motive, the object towards which the action is directed, the determined set of operations that act and the model according to which the subject acts. The action constitutes the act of the subject’s vital activity. Finally, the action, like the activity, is subjective; that is, it belongs to the subject and always participates as an activity of a concrete personality (Talyzina, 2009, p. 16).

Actions are seen as processes directed towards a goal resulting from the historical development of the person, who is part of a society organized by work. It is observed in primitive divisions of labor, that the partial results achieved do not satisfy particular needs, but are satisfied by their participation in the product of common activity obtained through social relations (Leontiev, 1978, 2009).

The proposal to study human activity, the person’s relationship with the environment, is not simply a change of nomenclature, in which psychological functions are exchanged for psychological activity. The change is at the level of theoretical understanding, in which “the real process of human interactions with the world” is analyzed without working with isolated elements (Talyzina, 2009, p. 15).

It is from this initial relationship with the world of things and the world of people that personality develops. Over time, reality expands, moving from the narrow circle of people and objects around them to knowable and representable reality. “The real ‘field’ that now determines its actions is not simply the present, but the existing one, which exists objectively or at times only in an illusory form” (Leontiev, 1978, p. 163). Thus, personality formation is a process with no end, consisting of several stages whose qualitative particularities result from concrete conditions and circumstances (Leontiev, 1978).

It is worthwhile here to clarify parenthetically that it is not just any type of contact between the adult and the child that promotes development. This adult-child relationship must go beyond the social and contextual aspects, which refer to social learning, and should be considered the acquisition of human experience — the psychological tools accumulated throughout history within a given culture (Quintanar & Solovieva, 2017). In this sense, Vygotsky argues, based on the periodization of child development, that for each psychological age, there is a central formation and accessory formations that lead to psychological development (Vygotsky, 2006).

Things presented to children by adults acquire functional meaning. Thus the “objectified activity of the child acquires a tool structure, so much so that communication becomes verbal, through language” (Leontiev, 1978, p.161).

Finally, we conclude that “the object of psychological analysis is not the psyche as such, but the activity, whose elements can be external, material, and internal, psychic” (Talyzina, 2009, p.22).

Next, we reflect on Activity Theory’s contributions to understanding the preschool stage.

### Role-Playing as the Main Activity of the Preschool Phase

At each age, there is always a central formation as a guide for the whole process, and there are also partial processes, which are accessory lines of development. These central and partial lines alternate with changing ages; each stage has its own structure (Vygotsky, 2006).

The child is capable of performing various activities in specific social situations. Still, there is a main activity that will boost psychological development at that particular stage of the child’s life. Through this activity, the child acquires new psychological aspects (Solovieva & Quintanar, 2012).

Therefore, the essence of each age is in the new formations, which are:

... the new type of structure of the personality and its activity, those mental and social changes that first appear at a given age and that mainly and basically determine the child’s consciousness, his relation to the environment, his internal and external life, the whole course of his development during a given period (Vygotsky, 2006, p. 254).

Each chronological/psychological age is favored by a specific social situation, in which the main activities need to be developed with the support, guidance, and conscious participation of the adult/educator. It is this that will promote the psychological development of the child (neoformations).

At preschool age, the organism is in intense development, and the gains in the physical aspect offer children greater independence. This will interfere with their relationship with the adult. That is: “joint activity is replaced by the independent fulfillment of the instructions given by the adult” (Mukhina, 1980, p. 56).

There is an increased awareness of one’s own “I” and one’s actions, and a growing interest in the world of adults and their activities. It is this need to know the adult world that leads to the game in its most developed form, i.e., role-playing as the main activity of the preschool phase, which allows the modeling in the child of social relations. An isolated action with an object has no meaning. It only acquires social meaning and real motivation when this action is part of human relations. This is possible in role-playing (Elkonin, 2009).

Therefore, the thematic social of role-playing will be a fertile field allowing the child to develop knowledge of social relationships and thereby acquire new psychological formations. “The orientation towards his colleagues, towards the opinion of the nascent community, forms the social sense in the little one: the spirit of initiative, the ability to follow the group, to share feelings, etc.” (Mukhina, 1980, p. 58).

Some studies conducted in Mexico and Colombia (Gárcia et al., 2013; González & Solovieva, 2014; González et al., 2011; Moraes, 2018; Solovieva et al., 2015) indicate the psychological development of children in preschool when using thematic social role-playing as the main activity.

In Brazil, we also have some studies (Andrade, 2017; Colussi, 2016; Colussi & Szymanski, 2020; Souza, 2010; Steinle, 2013; Vieira, 2017) that point to the use of role-playing in early childhood education as an important activity for the development of the creative imagination, of higher psychological functions, favoring the regulatory role of language, contributing to the children’s singularization process and the development of the voluntary activity.

At preschool age, role-playing occupies an important space in children’s development, allowing them to experience adult social life playfully. Social roles are experienced within the game, where rules are respected, and conflicts are managed according to the children’s abilities. This promotes role-playing as the main activity of preschool age (Venguer, 1976).

In light of this, Elkonin (1980) points out that:

... the game’s world has its rigid laws, which are reflections or copies of the real relations existing between people and objects or between one object and another. The game is not a world of fantasies and conventionalism, but a world of reality without conventionalism, reconstituted solely by unique ways (p. 212).

Given the theoretical and practical evidence of the importance of role-playing for child development, it is worth considering how teachers can organize and use it. Early childhood education’s task is to develop a pedagogical way to work with role-playing, focusing on social relationships. For that, we highlight some mediations that the teacher can perform, such as: “playing together with the children; reading stories about a theme that the child is playing with in their games; organizing a visit to one of the situations present in the child’s play, etc.” (Nascimento et al., 2009, p. 301).

Furthermore, it is important to disseminate a method proposed by Solovieva and Quintanar (2012), which was developed at Kepler College and has been used in several studies (Bonilla-Sanchez & Solovieva, 2016; Gárcia et al., 2013; González et al., 2011; González & Solovieva, 2014; Solovieva et al., 2015), with favorable results for child development.

This proposal can be developed with children from 3 to 6 years of age, as at this stage, there is an interest in adults’ actions and attitudes, and it can also be used with children who have developmental difficulties. This activity should be introduced gradually at the preschool stage. It starts with acting with concrete objects and then with substitute objects, until reaching the most developed way of playing social roles, which almost does not require using objects. The school must respect these steps when introducing role-playing, considering that the same theme can be developed differently, depending on the stage of the group (Solovieva & Quintanar, 2012).

A program for game activity in preschool institutions should consider the following steps: discuss and propose a theme; define and choose roles; analyze and define what each character does; analyze and define the means (objects) that will be used, and, finally, analyze the activity performed, highlighting who performed the role properly, what needs to be improved (Solovieva & Quintanar, 2012).

Observing and recording the children’s progress and the characteristics of their voluntary activity are important. It is worth mentioning advances such as: taking initiative to propose new themes and characters; new verbal and non-verbal actions, different from those defined together with the adult; reduction in the use of objects and dependence on materialization (Solovieva & Quintanar, 2012).

The role-playing game is structurally organized into three functional parts: planning/organization (guiding), execution, and control/reflection, according to the functional parts of every activity (Talyzina, 2009). The guiding part concerns the concrete conditions necessary for achievement of the action. Execution is the work in action, transforming the material or ideal object. The control part is the confrontation between the results obtained and the initial model, making necessary corrections in the guiding and executing parts (Talyzina, 1988).

The “action guiding base” is the theoretical and practical information that helps the child to perform the requested action. In role-playing game activity, “the guiding base represents much simpler and more accessible training for the child than school learning activity” (Solovieva & Quintanar, 2012, p. 67).

In role-playing, the teacher uses the action guiding base to distribute roles for verbal and non-verbal actions. The teacher can use different strategies to form verbal actions, depending on the children’s developmental level. At the beginning, the teacher can offer examples of words and phrases that can be used according to the social situation. “Children take up this verbal material by imitation, animation and help, in which the teacher initiates the word and the sentence, while the children continue and develop it” (Solovieva & Quintanar, 2012, p. 69).

On the other hand, the guide basis for the formation of non-verbal actions in the game includes several actions, but “initially it is about actions with objects and symbolic ones, which must be formed at the preschool stage. The more complex actions are gradually included by example, imitation or verbal suggestions from the adult” (Solovieva & Quintanar, 2012, p. 69). At a higher developmental level of role-playing activity, children follow their guidelines for performing verbal and non-verbal actions.

Finally, the thematic social role-playing game is not a pastime. It has objectives presented by the teacher and reasons that awaken the children to participate in the activity. Through this method, children improve coexistence, develop language, respect for others, flexibility in actions and thinking, and improve communication strategies, cooperation, and skills to resolve conflicts, develop imagination and self-regulation skills (González & Solovieva, 2014; Veraksa & Veresov, 2022).

Given the theoretical and practical evidence of this didactic strategy for developing preschool children, it is worth reflecting on teaching practice in Brazil.

### Teaching Practice in Brazilian Preschool

Most institutions dedicated to early childhood education face a welfarist past, as the first institutions designed for this age group emerged to serve poor children and especially children of working women. Education was more focused on moral issues, as the children needed to adapt, leaving aside intellectual issues (D. Saviani, 2012).

Over time, there has been a demand for these institutions, whose main objective is basic care, to offer quality care and time, that is, more comprehensive care. The new pedagogical ideas also presented information about child development, addressing the need for greater attention to childhood with specialized professionals. N. Saviani (2012) points out that private institutions adopted these transformations over time, although public ones did not advance in the same proportion.

The recognition of early childhood education as the first stage of basic education in Brazil, by the Law of Guidelines and Bases for National Education (Lei de Diretrizes e Bases para a Educação Nacional - LDBEN) (1996), was an important achievement. However, it was not enough to guarantee major transformations for two reasons: the non-mandatory nature of this school phase, and the idea that the preschool phase would not be configured as a stage of school education (N. Saviani, 2012).

The result is confusion, where we have, on the one hand, institutions that believe that the preschool stage needs to be a free, informal space with a curriculum built by the children themselves (Prado & Azevedo, 2012), and on the other, we find early childhood education that suffers from premature literacy: “for the child to learn, in fact, to read, write and deal with numbers – with understanding, resourcefulness, and autonomy – much has to be done, in the formation of their mental processes” (N. Saviani, 2012, p. 70). However, we need to overcome the worldwide trend of focusing on the cognitive development of young children with practices distanced from play (Fleer, 2022).

Arce (2013) credits part of this confusion to Brazil’s National Curriculum Guidelines for Early Childhood Education (Diretrizes Curriculares Nacionais para a Educação Infantil), which presents two guiding axes, i.e., interactions and games that are not defined and explored in the document. In practice, “Early Childhood Education is not school” (Arce, p.18) and, therefore, children need the freedom to play and interact among themselves without adult intervention to curb their creativity.

Although the premise “playing + freedom = happy child” is entangled with the definition of what it means to be a child and childhood in our country, it is incomplete, not contributing to the knowledge of who a child really is and, much less, so that the pedagogical work presents itself as something efficient, generating development in Early Childhood Education (Arce, 2013, p.18).

It is necessary to clarify that the teacher has a fundamental role in early childhood education as a mediator of cultural products constructed throughout history. Therefore, the child needs to have the opportunity to:

explore objects through manipulation, and understand their function and social utility, such as appropriating literature in moments of storytelling or even playing social role-playing, among others. Thus, the greater this involvement is, the greater their cultural appropriation and objectification process and, therefore, the more qualitative their imaginative and creative activity will be (Steinle, 2013, p. 115).

Silva and Lima (2015), in a survey conducted at an early childhood education school, found through observations and interviews that teachers recognized playing as an important part of children’s development. Thus, it should be part of the routine, without intervention from the adult. They also found that interventions by the teachers were restricted to situations of conflict. The same was observed by Singer et al. (2014) in a survey of Dutch nurseries, where teachers spend most of their time moving around without getting involved in the children’s play, and this has a negative impact.

In this way, most of the games were free, without the teacher’s intervention, and there were also games proposed by the teachers that did not always work due to complex rules that left the children disinterested. However, it is necessary “to recognize play as a tool that should be used not only to distract the child or occupy him, but to allow him to advance in his development through enriching experiences” (Silva & Lima, 2015, p. 61). It is worth mentioning that

…at the institution, it is easy to identify how much children already play using the experiences acquired in other social relationships. Yet, we want to emphasize the importance of playing, which can further contribute to child development, mediated by the teacher (Silva & Lima, 2015, p. 63).

Thus, the importance of playing and the time allocated for it depends not only on the schools’ curricula, but also and mainly on the training of teachers who work with early childhood education. Specific theoretical knowledge of psychological development, pedagogical knowledge of content, didactics, and methodologies must be indicated for each age group.

This knowledge needs to reach the schools of early childhood education, and this is the goal of the internship for training psychologists, which I coordinate at a public university in the central-west region of Brazil. In the next section, we report on an experience with positive results.

### Activity Theory Applied to Preschool Through Internships for Professional Training in Psychology

Vygotsky sees education, in a broad sense of intersubjective social practice and not necessarily dependent on a structure, as essential for individual psychological development and the subject’s personality. A great leap in development is linked to the human capacity to create and risk following other paths (González, 2007).

From this perspective, teachers play an active role in the teaching and learning processes, as it is up to them to promote all children’s development. A contrary stance would be to identify cognitive skills that previously existed in the child and ensure external conditions for them to develop. This approach is the cause of many school failures, because if teachers believe that a student is born with cognitive abilities, then those with difficulties would also have innate cognitive problems and, thus, be unable to learn. So the teacher would have nothing to do (Talyzina, 2009).

Given this,

[t]he intentional and planned influence of teaching on personality formation has been very small, and, among the reasons for this, we highlight both the conception of personality grounded in traditional psychology and the consequent lack of knowledge about its development, which includes the recognition of the most powerful experiences for its formation at the different age stages (Martins, 2006, p. 27).

School failure and learning problems are common situations that interfere with the lives of many children. Nevertheless, little or nothing is done to resolve them; most of the time, they are accepted as simply facts. Sometimes, administrative changes are made to school contents for each subject, insertion and/or removal of subjects, and age group changes according to the school year, but “they do not propose specific alternatives that make it possible to solve this problem or reconsider programs and teaching methods” (Solovieva & Quintanar, 2009, p. 7; Talyzina, 2009).

In this sense, Vieira (2017) warns that:

... our capitalist society increasingly encourages meritocracy, punishments, individual stimuli and incentives and all the bourgeois behaviors that Vygotsky tried to combat. We form individualistic, selfish people, incapable of postponing satisfaction and controlling impulses, with all these characteristics present in the so-called “learning problems” for which students are blamed, disregarding that they are formed by production relations (p.61).

Children who have low development of the voluntary self-regulation when they reach school age^1^ are subject to the following complaints from the teacher: they do not include themselves in the group activity in the room, do not follow the teacher’s instructions, are often distracted, do not complete tasks in the allocated time, cannot organize notebooks, are impulsive, uninhibited or passive and dependent (Salmina & Filimonova, 2001).

These are the complaints we received in the internship in school psychology, developed in schools of early childhood education and elementary education of a public university in the central-west region of Brazil. Therefore, there is a need to find a path that does not hold children responsible for their difficulties at school, but rather promotes the development of psychological neoformations that can contribute to their success.

In 2017, the author of this text completed a sandwich doctorate (Program of Sandwich Doctorate Abroad, PDSE), funded by the Coordination for the Improvement of Higher Education Personnel-Brazil (Coordenação de Aperfeiçoamento de Pessoal de Nível Superior-Brasil — CAPES). In a master’s program in Diagnosis and Neuropsychological Rehabilitation at the Benemérita Universidad Autónoma de Puebla in Mexico, it was possible to participate in courses under the supervision of Prof. Dr. Yulia Solovieva. Part of the data from a doctoral research project (Moraes, 2018) was collected at Colegio Kepler, where role-playing is one of the main foci of the work developed by teachers of children from 3 to 6 years old. The teachers used the methodology for introducing role-playing by stages in early childhood education developed by Solovieva and Quintanar (2012).

In the last five years, internship supervisions have been carried out in early childhood education based on Activity Theory, with positive results. *Table 1* summarizes the adaptations made to ensure the involvement of the class with the role-playing and the evolution in the development of the game level.

The program presented above comprised 12 sessions, which already resulted in advances in the game level. The class teacher identified changes in the children’s actions at other times in the class, such as greater control of behavior, greater understanding of the instructions for the proposed activities, and respect for the rules (Sousa, 2023).

**Table 1:** Introduction of role-playing in a Brazilian public school

Steps	Early games (1st to 4th)	Intermediate games (5th to 9th)	Final games (10th to 12th)
**Planning** Theme Choice	The adult suggested the theme (most common — restaurant). Introduction of the topic using a child´s video representing the social situation; Students found it difficult to talk about the topic.	Students discuss interests and preferences — restaurant game turned pizzeria at the students´ request. The adult suggested new themes: vaccine room, market, and pet shop.	Students chose the ice cream store theme by themselves. The student who suggested the idea felt important when the adult and the group accepted the suggestion.
**Planning** Presentation and Division of Roles	The mediator adult uses two-option questions: Who works at the restaurant: the fireman or the waiter? Students had difficulties thinking about roles. The number of students in the class (30 children) made dividing the roles difficult.	Students can list the roles according to the theme of the game. Students need clothes, accessories, and tools that characterize the characters. Division of the group, through verbal agreement, into two sub-groups (characters and observers); Children have difficulty staying in the previously divided role.	Students can list the roles and suggest new roles during the game. Division of the group between observers and characters using a marking with colored ink on the hand; There has been an increase in staying on the role until the end of the game.
**Planning** Symbol Construction	The adult constructed less complex symbols (e.g., cardboard with an option) without the children’s participation. Children’s difficulty in following the guidelines of the symbols.	Symbols defined and built together with the children; Symbols with more elements facilitated the child’s verbal and non-verbal actions. Children were encouraged to construct the symbols but were unsuccessful because of the short time set for the games.	Children use easily constructed symbols.
**Planning** Rules De$nition	Rules defined by the adult with the children´s participation; Difficulties following the rules due to not understanding the game’s purpose.	Rules defined together with the children; Need to build new rules after the first round and time for reflection; Symbolization of the rules on the board.	Internalization of rules and performance according to what was defined in the group.
**Planning** Choice of Objects	Attachment to the objects defined for roleplaying; Child chooses the character according to the work object. The adult presents the objects at the beginning of the game.	Students are unaware of the social utility of some objects. Necessity of resuming the object game with new themes (vaccine room, pet shop).	Students use the objects according to their social function. There is still an attachment to objects for the game realization. How ever, this has boosted the game’s development. The children are capable of using substitute objects.
**Game Execution**	Children have difficulty staying in their social roles. Children are distracted by objects. They do not understand the purpose of the game. They have difficulty playing with the group.	Increased awareness of the purpose of the game; Children develop verbal and non-verbal actions according to the theme, supported by objects and symbols. They get excited when the intern arrives at the start of the game.	Children understand the need to remain in the role until the end of the round. The game develops with greater autonomy on the part of the children and less interference from the adult. The children suggest new themes, characters and verbal and nonverbal actions. Increased cooperation among peers.
**Reflection and Control**	Students resist participating in reflection and control. Get distracted easily.	They participate by answering the mediating questions (What was the game today? How did the waiter serve the customers?)	Students took advantage of the role of observers to point out mistakes while the game was taking place. Students identify failures more easily and correct their peers. They can identify mistakes and successes.

*Note: Data taken from Ana Beatriz Oliveira de Sousa’s Course Completion Work (2023) built through an experience report in an internship and extension project, with role-playing developed with a group of 5- to 6-year-olds.*

Given the positive results, the goal is to continue with the interventions with more sessions and involve the class teacher in the process so that she can continue the work. It is also proposed to organize a training course for early childhood education teachers based on the experiences developed in the municipality, so that Activity Theory applied to preschool can be used by teachers and included in the education system in the future.

## Final Considerations

We achieved the article’s objective by highlighting N.F. Talyzina’s methodology applied to the preschool stage, as shown by the favorable development records when the teacher systematized and organized students’ activity. It is up to the teacher to understand that role-playing is the main activity that leverages the development of children between 3 and 6 years old and that it must be worked on at school through the action’s guiding base.

We advocate the continuing training of teachers of early childhood education from the perspective of Historical-Cultural Theory and Activity Theory, so that they can contribute to the transformation of pedagogical practice with actions that ensure the approach of children with the tools of culture, with the provision of diversified materials, and with planned teaching considering the knowledge accumulated by humanity.

Finally, there is a need to expand formative experiments to the Brazilian population, with scientific dissemination of the results.

## Footnotes

1 We consider school age to be the period a(er the age of 6, when the child leaves early childhood education and goes to Elementary School I (Talyzina, 2009).
